# Regional Integrated Cardiovascular Risk Management Care Pathway in the Netherlands: Benefits and Working Mechanisms

**DOI:** 10.5334/ijic.8654

**Published:** 2025-07-01

**Authors:** Relinde J. de Koeijer, Marcella E. de Geest, Gideon R. Hajer, Fabrice M. A. C. Martens

**Affiliations:** 1 Institute of Health Policy and Management, Erasmus University Rotterdam, PO Box 1738, 3000 DR Rotterdam, NL; 2 Centre for Cardiovascular Disease Salland, Deventer Hospital, Deventer, NL; 3 Department of Internal Medicine and Centre for Cardiovascular Disease Salland, Deventer Hospital, Deventer, NL; 4 Department of Cardiology, Deventer Hospital, Deventer, NL; 5 Department of Cardiology, Amsterdam University Medical Center, Amsterdam, NL

**Keywords:** Cardiovascular Risk Management, care pathway, integrated care, evaluation, benefits, working mechanisms

## Abstract

**Background:**

Integrated cardiovascular risk management care pathways are initiated nationwide to decrease the morbidity, mortality, and costs of cardiovascular diseases. However, evidence on cardiovascular risk management care pathways is needed to support broader scaling up of these initiatives.

**Aim:**

To evaluate a Dutch cardiovascular risk management care pathway to identify benefits for patients and professionals and determine working mechanisms for upscaling integrated care initiatives.

**Methods:**

Process and clinical indicators: retrospective cohort design combined with quasi-experimental time-series design with longitudinal data (2014 to 2019, n = 3779) examined using an ANOVA with contrasts. Team indicator: a survey. Working mechanisms: multidisciplinary focus groups and interviews with patients.

**Results:**

Process, team, and clinical indicators showed that cardiovascular risk management care pathway is beneficial for enhancing quality of care and inter-professional collaboration. Six working mechanisms were identified: boundary spanners, boundary objects, network platform, continuous learning and improvement, mixed-methods evaluation, and multilevel connection for upscaling.

**Conclusion:**

Vertical integration formalized in a care pathway benefits both patients and professionals in primary and secondary care settings. Also, evaluation with multiple research methods enables a more comprehensive understanding of the context in which care pathways are implemented and working mechanisms, which is essential for scaling up integrated care initiatives.

## Introduction

Cardiovascular diseases (CVDs) may result in an increase in chronic conditions, mortality, and healthcare costs, thus, burdening healthcare systems [1,2]. People diagnosed with CVD experience barriers to receiving timely care, in part because healthcare systems and care processes are not designed to wholly meet the needs, expectations, and characteristics of this specific patient group [3], and other chronic conditions. Furthermore, patients often receive protocolized and fragmented care from different disciplines in primary and secondary care [4]. During the last decade, clinical practice and research have focused on integrated care models as a solution to build more effective and efficient healthcare systems that better meet the needs of patients [5]. Many different definitions of integrated care are used in the scientific literature and in practice [6]. In accordance with the World Health Organization (WHO), we define integrated care as:

“An approach to strengthen people-centered health systems through the promotion of the comprehensive delivery of quality services across the life-course, designed according to the multidimensional needs of the population and the individual and delivered by a coordinated multidisciplinary team of providers working across settings and levels of care” [7].

Care pathways can be seen as vertical integration across primary and hospital care services [8,9], for a variety of patient groups with chronic conditions, including CVDs [10]. Several studies conducted in Europe demonstrate benefits of care pathways for both CVD patients and health care professionals, often reporting decreased practice variation and improved teamwork [11,12]. Nonetheless, the effectiveness of these care pathways remains controversial [13]. For example, several challenges emerge when conducting research related to care pathways, including longitudinal evaluations [14], combining improvements in processes (such as multidisciplinary teams) with improvements in clinical outcomes [15], and including a sufficiently large range of data for measuring integration, such as indicators derived from administrative databases [13]. Therefore, **the first aim** of this study is to evaluate a Dutch hospital’s integrated CVRM care pathway by investigating process (number of referrals), team (adoption, implementation, and control of CVRM care) and clinical outcomes (degree of achievement of target levels for LDL cholesterol levels and blood pressure levels) in a longitudinal manner**. The second aim** of this research is to identify working mechanisms for integrated care. It is essential to recognize the role of contextual variables in adapting the findings of a study to one’s own situation and specific patient population [16]. Moreover, single integrated care initiatives often succeed, but a broader transition and dissemination of these initiatives is difficult to realize [17,18]. A recent systematic review exploring transitions in healthcare found that there is a scarcity of literature pertaining to which factors determine success [19]. Accordingly, greater insight into the working mechanisms of integrated care is required to support broader scaling up of these initiatives [7,8,20].

## Methods

Care pathways consist of components that can work both independently and interdependently [21,22,23]. For care evaluations, summative or formative methods are commonly chosen [24]. Formative evaluation methods are suitable for this research as we aim to evaluate a CVRM care pathway and identify working mechanisms for expanding integrated care initiatives. According to the ethics committee of Isala Zwolle, this study does not fall under the Medical Research Involving Human Subjects Act (WMO) and was therefore not subject to WMO obligations.

### Study design

To measure process, and clinical indicators, related to the first aim of our research, we utilized a retrospective cohort design over the period of 2014 to 2019. Data from 2020 and 2021 was excluded due to the COVID-19 pandemic. Using data from a six-year period is novel, given that relatively few integrated care initiatives have expanded beyond the initial pilot phase [8,20]. Data were extracted from the Electronic Patient Records (EPD) of the participating hospital. Thereupon, data selection and data validation meetings were held with stakeholders to select and validate the pseudonymized patient data (n = 3779). As no control variable exceeded an effect size of 0.30 [25], none of the control variables were entered in the analysis. We completed a quasi-experimental time-series analysis using Analysis of Variance (ANOVA) with contrasts. We differentiated groups by age in the analyses when relevant, given differences in target levels of LDL and blood pressure in various age groups. SPSS software was used for all analyses.

To measure the team indicator, also related to the first aim of our research, we conducted a survey of general practitioners (GP’s) and allied general practitioners professionals (sample size of 130 respondents) to determine the degree of adoption of guidelines, using the aspects of Adoption, Implementation and Control from the RE-AIM framework. The aspects were measured on a five-point scale (from strongly disagree to strongly agree) (Table 1). We used validated measurement instruments, such as the construct for learning and development climate from Patterson [26] on organizational climate. A part of the questionnaire is developed specifically for this study. Stability was determined for each scale. To examine the underlying structure of the instruments, a Kaiser-Meyer-Olkin (KMO) and Bartlett test was conducted. Item commonalities with values from 0.40 to 0.70 are accepted in social sciences [27]. Therefore, we excluded items with factor loadings lower than 0.50. Cronbach’s alpha was used to determine reliability. Based on a review of the literature, Taber [28] concludes that a value of 0.70 or higher is generally considered a sufficient measure of an instrument’s reliability or internal consistency. Therefore, we excluded items with a value lower than 0.70.

**Table 1:** Questionnaire Team indicator – Adoption, Implementation and Control.


CONCEPTS	ITEMS (FIVE-POINT SCALE)

Adoption and implementation	

The willingness of teams and healthcare professionals to adhere to integrated CVRM working practices	I am convinced that effective CVRM care is provided to patients in my practice. Within my practice, the willingness to work according to the CVRM guideline* is high. I feel well equipped to provide care to CVRM patients. Within my practice, there is positivity about CVRM care.

The degree of adherence of healthcare professionals with the Dutch guideline CVRM	I am familiar with the Dutch guideline CVRM. Within my practice, the Dutch guideline CVRM is followed faithfully. I find the Dutch guideline CVRM clear. Within my practice, the Dutch guideline CVRM is implemented as intended. If I notice that a colleague is not following the Dutch guideline CVRM, I discuss it.

Control	

The degree of collaboration, transparency in communication and coordination in an integrated setting	Within my team it is clear who does what for CVRM-patients. I have a clear picture of how CVRM tasks and patient-responsibilities are divided between primary and secondary care. Coordination of CVRM patients with Deventer hospital is effective. I know the route for contact/consultation with Deventer hospital well (e.g. VIPlive consultation or visits from the advanced nurse practitioner). It is clear which CVRM patients should and should not be referred to Deventer hospital.

The extent of continuous learning and improvement	Within my practice: … bottlenecks in CVRM care are quickly addressed. … required knowledge and experience is available to provide good care to CVRM patients. … problems within CVRM care are solved adequately. … colleagues are always looking for new, innovative ways to improve CVRM care

Other	At which GP practice are you employed? Open text area for extra information at the end of the questionnaire

*CVRM guideline	*Dutch guideline CVRM (2019) that has been endorsed by several societies (e.g. the Dutch Society of General Practitioners)*.


During three focus groups, we used contemplative dialogues to validate, enrich, and place in context the outcomes from the quantitative analyses and questionnaire, and in light of our second research aim to explore working mechanisms and insights for expanding integrated care initiatives (**Appendix 1**). Physicians, advanced nurse practitioners, a business manager from Deventer hospital, a GP, three allied GP professionals, and a policy officer from the Cooperation of General Practitioners (CGP) participated in the focus groups. We also carried out semi-structured interviews with four patients to better understand their perspectives and experiences in the evaluation. These interviews can provide insight into potential areas of care coordination and care continuity after discharge from hospital. All interviews lasted 1–1,5 hours, were partly online and partly in-person, and were transcribed verbatim. The focus groups and interviews were recorded (informed consent was provided) and transcribed. Two of the authors (De Koeijer & De Geest) reviewed the transcripts independently, using open codes to mark emergent key ideas and themes. The main author (De Koeijer) then clustered the data in more general categories, using the process of axial coding, and carefully selected illustrative quotes [29].

### Research setting

Deventer hospital is a medium-sized teaching hospital in the Netherlands with 371 beds, 220,000 outpatient visits, 2,400 employees, including 205 medical specialists. The GPs are united within a Co-operation of General Practitioners (CGP). Around 150 general practitioners in the region are members of this co-operative association. Deventer hospital and CGP collaborate within a network platform, named Centre for Cardiovascular Disease Salland (CCDS). Approximately 900 follow-up patients and 650 new patients receive care every year in the CVRM care pathway. Healthcare professionals involved in CVRM care include GP’s, advanced nurse practitioners, and allied GP professionals and physicians. Since its implementation in 2015, the CRVM care pathway has continually been enhanced to further optimize patient care, corresponding with the phases of the Development Model for Integrated Care (Table 2) [30]. Figures 1a and 1b visualize how the care pathway has been shaped since the end of 2019.

**Table 2:** Developments in the CVRM care pathway 2014–2019.


PHASE	YEAR	DESCRIPTION OF DEVELOPMENTS

Initiative and design phase	2014	*From cardiac lipid clinic to CVRM clinic*: Until 2014, there was a lipid clinic at Deventer Hospital. In 2014, the outpatient clinic was expanded, which included multiple aspects in addition to lipids in the context of preventing or reducing cardiovascular disease events. *New roles:* in July internist vascular physician joined Deventer Hospital and, in the fall, the internal training to become an advanced nurse practitioner was started.

	2015	*Executive Board decides on vascular care/CVRM program*: This decision mend the official start of CVRM care pathway. *Integrated working agreements CVRM put on paper*: By expanding the outpatient clinic (lipid clinic to CVRM clinic), the working agreements for those involved have been put on paper, including financial implications. *Ezetimib registered*: the drug is a cholesterol absorption inhibitor (has a lowering effect) and has especially great influence on LDL levels. This drug must be taken alongside PCSK9 inhibitors (additive to statin). *Prescribing PCSK9:* This is medication (injection) that lowers LDL. This drug is only indicated if the patient is statin (medication: cholesterol synthesis inhibitor) intolerant and has a very high risk of cardiovascular disease or has experienced re-event or 1 event and has diabetes or familial hypercholesterolemia. *Starting colleagues*: In Sep, two advanced nurse practitioners started training and advanced nurse practitioners from Vascular Surgery started. Secretarial support was also implemented.

Experimental and execution phase	2016	*Pre-eclampsia care at the CVRM outpatient clinic*: Another target group, namely ex-pregnants at high risk of cardiovascular disease, were referred to the CVRM outpatient clinic. This is primary prevention, and the patient is seen once. *Changes of colleagues*: in the autumn an advanced nurse practitioner (from Internal Medicine) started and there was a change of chief physician.

	2017	Advanced nurse practitioners started in the fall from cardiology and neurology at the CVRM outpatient clinic

	2018	The referral structure has been modified (directly to the outpatient clinic)

Expansion and monitoring phase	2019	*New (FMS/NHG) guideline CVRM (revision of 2011):* The LDL target levels have been equalized between GPC and Deventer hospital by this guideline. Previously, the Deventer hospital maintained stricter target levels compared to the GPC. *Diagnostics, namely transition from calculated LDL levels to measured LDL levels*: Previously, LDL was calculated by using the Friedewald formula. From this point on, most LDL levels could be measured, only some of the LDL levels continue to be calculated. *New colleagues and working method:* Advanced nurse practitioners start, a policy officer starts at the CVRM clinic, and GPs can digitally consult the advanced nurse practitioners at Deventer hospital. Yearly practice visits by the advanced nurse practitioner with the GP and/or allied GP professionals are initiated, with room for exchanging knowledge, discussing cases, training and seeing patients together.


**Figure 1a:**
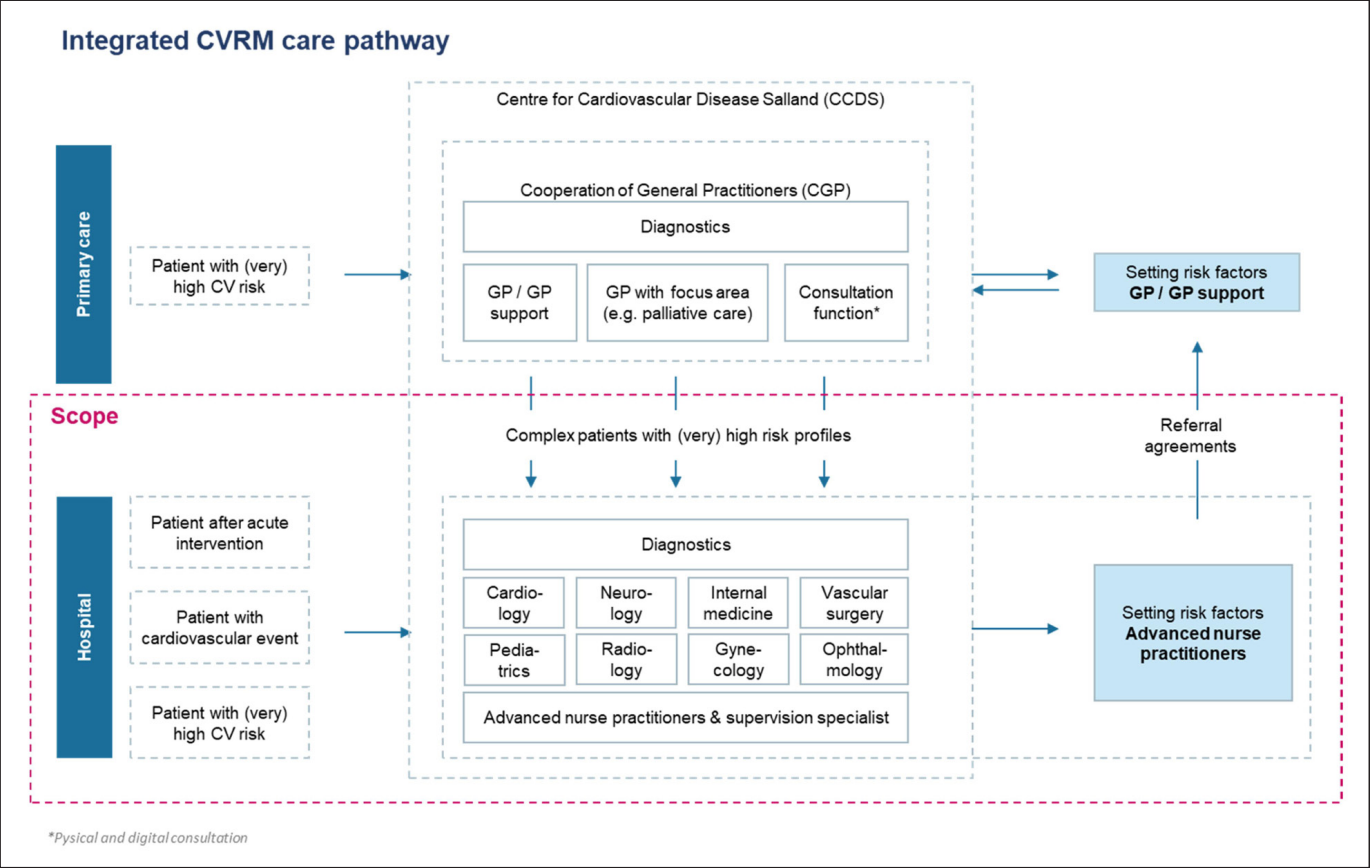
Integrated CVRM care pathway.

**Figure 1b:**
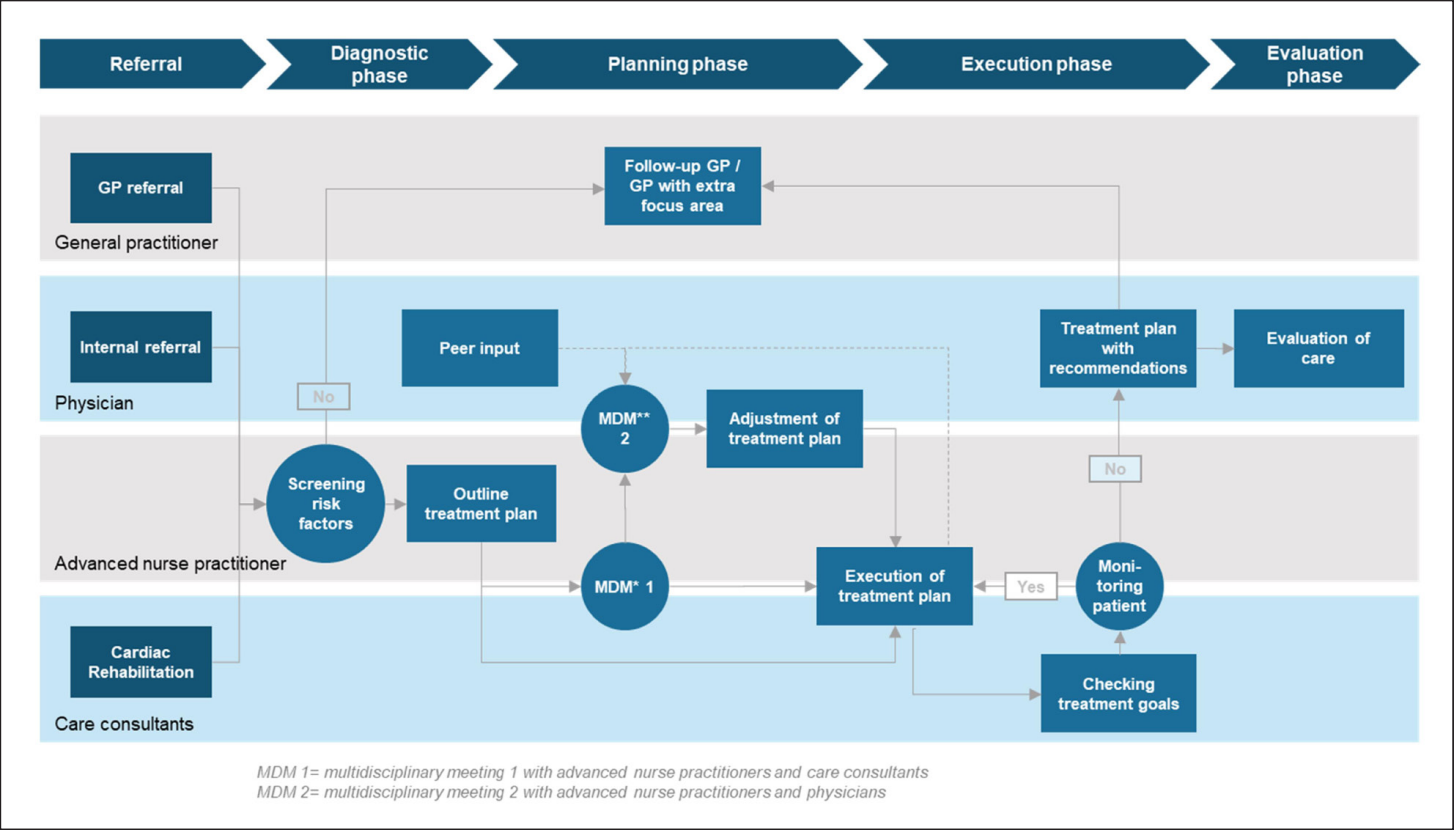
CVRM care pathway within Deventer hospital.

Many essential components noted in existing literature are implemented in this research setting, including regular comprehensive assessment of patients, multidisciplinary, coordinated teams, implementation of evidence-based medicine, training members of multidisciplinary teams, developing a consultation system, and exchange of patient information (with consent from the patient) between different care providers [31]. Notably, in this care pathway, complex CVRM care issues that emerge during the treatment period can be discussed in the weekly multidisciplinary consultation (MDC), and subsequently, required adjustments to the treatment plan can be made. Also, uniformity has been achieved between Deventer hospital and CGP, for example, through equalized LDL target levels, standardized work practices, and integrated working agreements. Finally, the advanced nurse practitioner functions as an important liaison between Deventer hospital and the CGP.

## Results

Patients included in our research were, on average, 64 years old and received, on average, 14 months of care in Deventer hospital during three appointments before being referred back to primary care.

### Process indicator

The number of follow-up patients in the CVRM care pathway has more than doubled in six years, from 399 patients in 2014 to 898 patients in 2019 (Figure 2a). A similar pattern can be seen for new patients, though the growth, compared to follow-up patients, is more gradually (increase of 384 new patients from 2014 to 2018) and a decrease can be seen since 2019, resulting in a lower number of new patients in that year than in 2016 (Figure 2a). From 2014 to 2019, there were 984 external referrals, with most being made by GPs. The number of referrals were relatively greater in 2015 and 2016 (119 and 114 external referrals, respectively). Growth continued in the following years by a factor of 2.45, to 279 external referrals in 2019 (Figure 2b).

**Figure 2a:**
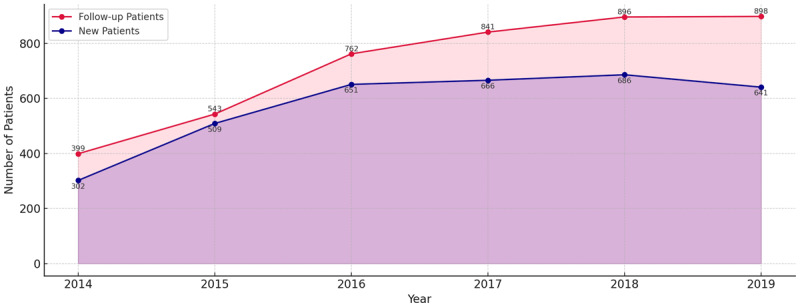
New patients and returning patients CVRM care pathway.

**Figure 2b:**
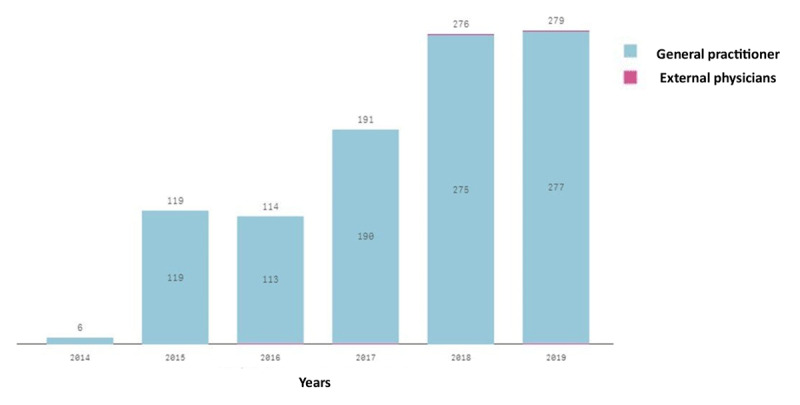
External referrals from patients to Deventer Hospital.

**Figure 2c:**
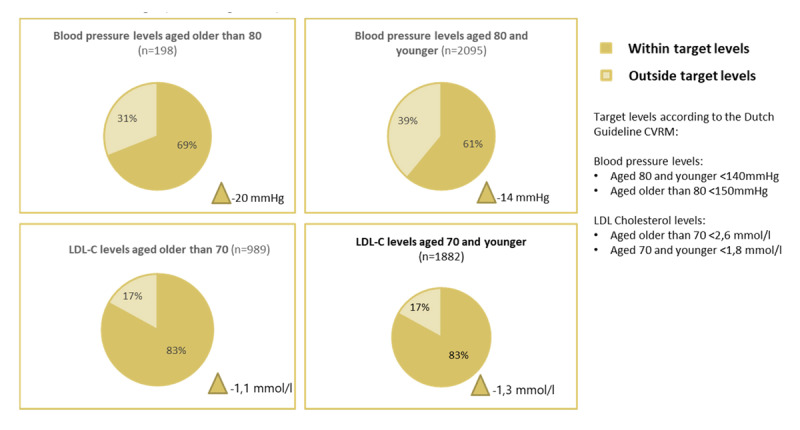
Average percentage patients within target levels according to the Dutch Guideline CVRM at discharge (including delta).

### Team indicator

The questionnaire, which measured the team indicator, was completed in June 2022 by 44 respondents. The respondents of the survey were distributed among 30 GP practices. This response rate of 34% is acceptable [32], and the psychometric characteristics of the measurement instrument are appropriate (Table 3).

**Table 3:** Psychometric characteristics of the measurement instrument.


		n	NO. OF ITEMS	µ	σ	CHRON-BACH’S α	KMO STATISTICS

A	Adoption and implementation						

	The degree of adherence to integrated CVRM working practices and Dutch guideline CVRM	44	9	4,11	0,43	0,83	0,74

B	Control						

	The degree of collaboration in an integrated setting and continuous learning and improvement within CVRM care pathway	44	9	3,87	0,10	0,81	0,73


For *Adoption* and *Implementation*, more than 90% of the respondents indicated that they feel well equipped to provide CVRM care and that Dutch CVRM guidelines have been well adopted.

“(…) boundaries – between professionals and organizations – are blurring” (advanced nurse practitioner Deventer hospital).

In the questionnaire, respondents were distinctly positive about the route of contact/consultation with Deventer hospital (such as digital consultation or visits of the advanced nurse practitioner). During the focus group, one of the allied GP professionals described it as follows:

“The digital consultation is very handy, to determine whether we really need to refer a patient. Often the digital consultation is enough to confirm that we still can do something in our GP practice and that we don’t have to refer a patient.”

Results regarding *Control* of CVRM care showed a more varied picture. Only 53% of respondents indicated that they had a clear picture of how tasks and responsibilities between primary care and Deventer hospital are divided for CVRM care. A physician from the Deventer hospital indicated that “We need to better communicate to the patient why he/she should go back for his/her pacemaker to the hospital but has to go to the GP for other CVRM care”. Although this is common practice in the Netherlands, the ambiguity also impacts patients. One patient stated: “They sent me for a checkup in the hospital while I just had been for a checkup at the GP.”

Finally, *Control* of CVRM care is also focused on the extent of continuous learning and improvement. 55% of respondents indicated that their colleagues were consistently exploring novel, innovative ways to improve CVRM care, citing insufficient time as the main obstacle.

“I am convinced that CVRM care could improve (e.g. more prevention), however, we are always in a time squeeze” (allied GP professional).

During the focus groups, participants mention that continuous learning and improvement was essential in developing the care pathway. During the 6-year period, frequent meetings between Deventer hospital and primary care, organized in the Centre for Cardiovascular Disease Salland (CCDS), with dedicated roles and resources, were initiated to learn and further enhance the CVRM care pathway.

“A success factor was the shared belief in and support for integrated CVRM care in the region (GP)”.

**Figure 3:**
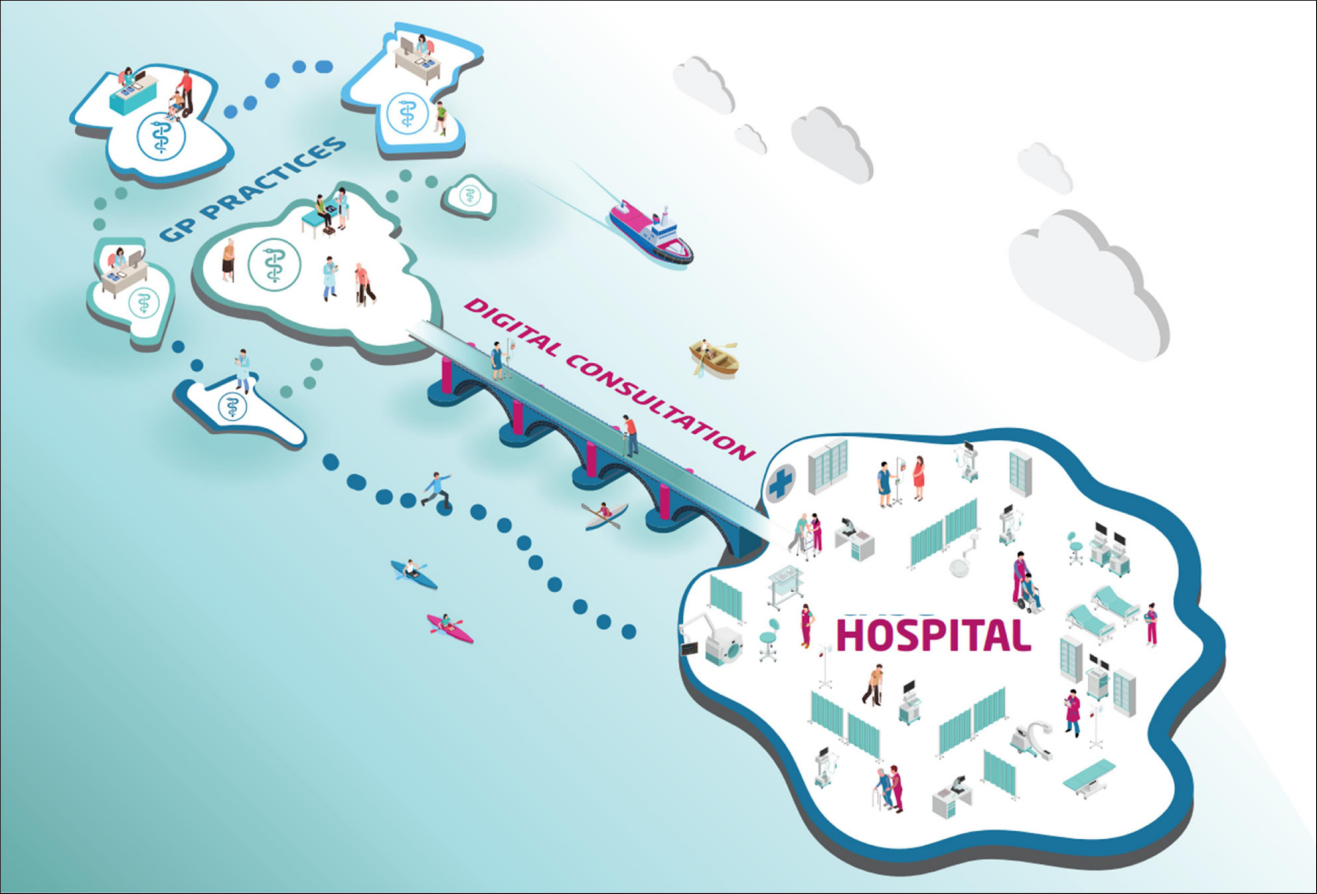
Visualization of ambitions for the future by primary care and Deventer hospital.

The desire to consistently improve CVRM care was expressed by many participants during the focus groups. Both primary care and Deventer hospital independently conceived the same vision for the future, focused on enhanced collaboration (Figure 3).

### Indicators blood pressure levels and LDL cholesterol levels

The mean blood pressure level at baseline (2014) was 145.8 mmHg and the mean LDL cholesterol level at baseline was 3.12 mmol/l. Sixty-nine percent of patients aged older than 80 and 61% of patients aged 80 and younger had blood pressure levels within target levels at discharge (<150 mmHg and <140 mmHg, respectively). In 83% of all patients, the LDL cholesterol level at discharge was within target levels (70 years and older: <2.6 mmol/l and 70 years and younger: <1.8 mmol/l). These rates are at the upper end of what has been previously published in past studies [33,34,35]. We found that the average difference between the baseline and discharge levels (delta) for both blood pressure levels and LDL cholesterol levels is promising (patients aged 80 and younger: –14 mmHg; patients aged older than 80: –20 mmHg; and patients 70 years and younger: –1.3 mmol/l; patients 70 years and older: –1.1 mmol/l) (Figure 2c). For example, we know from previous studies that each 1.0 mmol/l reduction in LDL-C is associated with a corresponding 20 to 25% reduction in mortality from cardiovascular disease and non-fatal myocardial infarction [36,37,38].

### Working mechanisms for integrated care

Our research showed four working mechanisms that supported scaling up the CVRM initiative from a CVRM clinic in 2014 to a regional integrated CVRM care pathway in 2019 (Table 2): three on professional level and one on organizational level.

#### Professional Level

First, nurse practitioners played a vital role by visiting GP practices, consulting for complex patients, and transferring knowledge between professionals in primary and secondary care.

“When physicians and GPs are involved, it becomes very much about the who should do what. Therefore, we (physicians) had to step back because collaboration between advanced nurse practitioners and primary care was realized much easier” (physician Deventer hospital).

Second, CVRM guidelines, and deliberating complex patient cases across organizations strengthened inter-professional collaboration and fostered stronger relationships between primary and secondary care. Third, our findings emphasized that continuous learning is essential in the scaling up off the CVRM care pathway.

“Frequently we reviewed what we have learnt over the past period, what those involved could handle and what the next step should be” (nurse practitioner Deventer Hospital).

Examples of this continuous learning include gradual trust-building, ongoing pathway evaluation, and collective goal-setting. In that sense, the ability to develop a capacity for learning can be identified as a working mechanism in shaping how diverse professional groups and organizations exchange knowledge.

#### Organizational Level

At the organizational level, the working mechanism we identified is related to the Centre of Cardiovascular Disease Salland (CCDS) that managed ongoing projects coordinated over 150 independent GPs, united in the CGP. Also, building trust between stakeholders was essential for the development of the care pathway through the years. The CCDS helped manage tensions, like GPs’ reluctance to refer patients, by formalizing agreements that include financial terms and are regularly reviewed.

“In the beginning, there was unfamiliarity, GPs were afraid that we were ‘steeling’ their patients. It took a lot of time and proving our worth before referrals increased (physician Deventer hospital)”.

In our study, Deventer hospital takes the lead in overseeing the CCDS, fostering collaboration across GP practices and the hospital.

## Discussion

In this study we evaluated a regional integrated CVRM care pathway in the Netherlands by investigating process, team, and clinical outcomes in a longitudinal manner (first research aim). Each of the indicators showed that CVRM care pathway is beneficial for enhancing quality of care and inter-professional collaboration, which is in concordance with earlier research [39,40,41,42]. Moreover, four working mechanisms were identified (second research aim), three on professional level and one on organizational level. Below, we discuss these working mechanisms in the light of scientific literature, and we propose two additional working mechanisms for future integrated care initiatives: one on system level and one methodological mechanism.

### Professional Level

First, we found that boundary spanners (in our case, advanced nurse practitioners) function as working mechanism since they play a crucial role in implementing integrated care pathways by connecting different groups and facilitating communication [43,44]. Two types of boundary spanners exist: those who do this as part of their main job, and those with a dedicated role [45,46]. In our study, advanced nurse practitioners perform boundary-spanning activities as part of their job. A recent meta-analysis [47] showed that less conventional roles such as pharmacists and community health workers are effective as well at leading blood pressure intervention implementation due to their expertise and boundary capabilities. Studies show that the role of boundary spanners is also relevant in other settings such as mental healthcare, ambulatory emergency care and community care [48].

Second, we found that CVRM guidelines, tailored for primary and secondary care, were the foundation for establishing shared understandings and facilitating collective learning [49]. Also, deliberating complex patient cases facilitated inter-professional collaboration [50] and fostered stronger relationships between primary and secondary care professionals. Ultimately, this process reduces gaps in coordination [51]. Both guidelines and complex patient cases appeared to be “both plastic enough to adapt to local needs and constraints of the several parties employing them, yet robust enough to maintain a common identity across sites” [52], allowing them to be characterized as “boundary objects”. These boundary objects function as working mechanism for implementing integrated care initiatives [51].

Third, our findings emphasized that integrated care is a dynamic, evolving process requiring continuous learning and improvement [15,53,54]. Integration can be viewed as a continuous learning process, with professionals and their organizations acting as learners within complex adaptive care systems [55], and the capacity for learning can be viewed as an essential working mechanism for knowledge exchange across boundaries.

The aforementioned working mechanisms on professional level highlight that skilled and motivated healthcare professionals who challenge boundaries [56,57] are key to developing effective care pathways, such as CVRM. However, the sustainability of such initiatives is fragile due to time constraints faced by professionals for further improvements. For that reason, it is also important to discuss mechanisms at organizational and system level.

### Organizational Level

To make integrated care initiatives more robust, they should be embedded in the broader context of the organization [58]. At the organizational level, the working mechanism we identified is having a network platform. In our study, the Centre of Cardiovascular Disease Salland (CCDS) can be characterized as a network platform since it is “an organization with dedicated competences, institutions and resources for facilitating the creation, adaption and success of multiple or ongoing collaborative projects” [59]. In the context of this research, the network platform provides a framework for various objectives that may evolve over time [60] serving as an important working mechanism in implementing integrated care initiatives.

### System Level

We also see untapped potential for the CCDS. It can mediate between local networks and national authorities, facilitating change beyond network boundaries at health-system levels [59,60,61]. This study focuses on the professional and organizational levels. However, as per past literature, at the *system level*, barriers for integrated care can arise. For example, Dutch healthcare professionals lack information systems capable of following the patient across different care settings. Also, pricing systems in Dutch health care systems lack adequate mechanisms to capture the contribution of cross-domains collaboration to deliver outcomes and value [62]. CCDS as a network platform can potentially connect professional, organizational, and system levels in the future, which may aid in further scaling up the integrated CVRM care pathway.

### Methodological Mechanism

Finally, our study highlights the importance of mixed methods to monitor integrated care as a methodological working mechanism. In addition to closely monitoring clinical indicators, we sought to understand the subjective narratives of professionals and patients. At the professional level, this is not new; in multidisciplinary meetings, patient cases are discussed in both a qualitative and quantitative manner. However, qualitative accounts may not be given the same value as objective clinical outcomes. With the current focus on quantitative measurement instruments such as PROMs and PREMS, the dominant view ‘to measure is to know’. However, the beneficial effects of these measurements have been overstated, and they have several potential negative effects including a narrow focus, a rise in unethical behavior, and reduced intrinsic motivation [63]. Evaluating integrated care initiatives also involves understanding the context in which they are implemented and identifying what is effective, the reasons behind their success, and for whom they are effective to increase the usability and applicability [4,11,64]. Qualitative data is required to achieve this aim. Accordingly, an essential working mechanism is to use mixed methods to monitor and evaluate integrated care initiatives.

## Practical Learning

Our study offers several practical insights for teams initiating integrated care pathways. First, a motivated group of professionals is essential to drive change. It is also important to monitor the balance between follow-up and new patients to ensure adequate care for all. Collaboration with GPs is key to ensuring appropriate patient referrals, and care pathways should remain adaptable to include new patient groups when necessary. Furthermore, uncertainty remains about discharging patients who have not met target levels; reflection on such decisions is crucial. In case of patients not reaching their targets despite of intensive treatment, mostly fragile elderly, their options are discussed in the multidisciplinary teams. Other (non) medical treatment options for reductions of cardiovascular risk are evaluated (including a more stringent target for feasible risk factor) or additional analysis for secondary causes are ordered. When patients do not reach their target levels, GP’s are informed about the reasons why we accepted different target levels. Evaluating care pathways can also reveal ways to save time and improve cost-effectiveness. For example, we found redundant LDL cholesterol tests and unclassified data entry in the electronic patient record (EPD). Addressing these inefficiencies can ease workloads and free time for preventive care. Data registration optimization is critical. Inconsistent entries, such as blood pressure recorded in various EPD locations, require manual checks. Finally, our research team was diverse in background, expertise, and age, contributing to a comprehensive multimethod study that provided a holistic view of the integrated CVRM care pathway.

## Study Limitations and Highlights

This study has several limitations. First, the number of interviews with patients in our research is limited and should be interpreted with caution. Future research could encompass alternative methods for gathering patient perspectives such as encouraging patient to keep a journal of their experiences, and thoughts over a period and shadowing patients throughout the care process and observing their interactions and reactions firsthand. Second, unfortunately, data from secondary and primary care were not integrated in the EPD and therefore, patient data could not be analyzed for the total care pathway. We initially tried to connect data from Deventer hospital with data from GP practices using a unique identification code. However, GP practices could only extract data for patients at the group level and not at the individual patient level. Third, data from Deventer hospital needed to be collected partly manually because of quality issues. Two team members of the research team reviewed the manually collected data independently, however, the possibility of errors remains. Fourth, most participants in the focus groups were involved with the care pathway from the beginning, and thus may have biased views; they were also initiators of the care pathway.

This study has several strengths. The longitudinal nature of the data, over a six-year period, is a unique aspect of the evaluation. Also, the use of a broad set of quantitative indicators (clinical, process, and team indicators) and qualitative data permit the identification of both the benefits of the integrated care initiative as well as working mechanisms, which is essential for expanding integrated care initiatives. In addition, we determined several practical considerations for teams who are planning to initiate an integrated care initiative. Finally, this study has contributed to further strengthening the collaboration between primary and secondary care within the Deventer hospital. In this regard, this study expedited the further implementation and scaling up of the integrated care initiative that was the subject of investigation.

## Conclusion

The findings of this study portray a positive outlook on the CVRM care pathway. The adoption and implementation of the Dutch CVRM guidelines increased satisfaction for all care-members and patients involved in the CVRM care pathway. This is supported by higher percentages of patients that achieved target levels, and greater differences between the baseline and discharge levels (delta) of blood pressure level and LDL cholesterol. Several working mechanisms were identified. At the professional level, our findings highlighted the crucial role of boundary spanners (in our case, advanced nurse practitioners) and boundary objects (in this study, patients and guidelines) in implementing integrated care initiatives. Also, continuous learning and improvement at the professional level is essential for making the integrated care pathway work. At the organizational level, we found that the role of the network platform CCDS was essential to cope with potential tensions between partners of the network. In addition, the network platform can potentially connect professional, organizational, and system levels in the future, which may help further expand the integrated care initiative. Finally, it is essential to consider both quantitative and qualitative data when implementing and monitoring integrated care initiatives. This facilitates the (automated) assessment of the advantages of integrated care initiatives, comprehension of the implementation context, and identification of operational mechanisms, all of which are crucial for scaling up integrated care initiatives.

## Appendix 1 – Contemplative dialogue

The conversation form is deduced from contemplative dialogue; a form of speaking and listening derived from the insights of Ignatius of Loyola, founder of the Jesuit order (16th century). The conversation form focuses on really listening to each other, wanting to understand each other, and using diverse perspectives. The form contains four steps:

1. *First personal reactions to the insights presented from data and questionnaire*. Each participant answers the following three questions:
  - What do I notice?
  - What am I curious about?
  - What do I learn from this for subsequent initiatives?
2. *Personal reflection*. Each participant writes down for him/herself and silently for 5 minutes what the reactions do to him/her and how he/she relates to them.
3. *Sharing reflections*. In random order, participants read to each other what he/she has written, without questions or reaction.
4. *Discussion on lessons learned and working mechanisms*. Participants discuss overarching learnings and working mechanisms regarding the implementation of integrated care.
